# The Metabolic Cost of Walking in healthy young and older adults – A Systematic Review and Meta Analysis

**DOI:** 10.1038/s41598-019-45602-4

**Published:** 2019-07-10

**Authors:** Sauvik Das Gupta, Maarten F. Bobbert, Dinant A. Kistemaker

**Affiliations:** 1 Department of Human Movement Sciences, Faculty of Behavioural and Movement Sciences Vrije Universiteit Amsterdam, Amsterdam Movement Sciences, Amsterdam, The Netherlands; 2 Human Movement Biomechanics Research Group, Department of Movement Sciences, Biomedical Sciences Group, KU Leuven, Leuven, Belgium

**Keywords:** Physiology, Ageing

## Abstract

The Metabolic Cost of Walking (*MCoW*) is an important variable of daily life that has been studied extensively. Several studies suggest that *MCoW* is higher in Older Adults (*OA*) than in Young Adults (*YA*). However, it is difficult to compare values across studies due to differences in the way *MCoW* was expressed, the units in which it was reported and the walking speed at which it was measured. To provide an overview of *MCoW* in *OA* and *YA* and to investigate the quantitative effect of age on *MCoW*, we have conducted a literature review and performed two meta-analyses. We extracted data on *MCoW* in healthy *YA* (18–41 years old) and healthy *OA* (≥59 years old) and calculated, if not already reported, the Gross (*GCoW*) and Net *MCoW* (*NCoW*) in J/kg/m. If studies reported *MCoW* measured at multiple speeds, we selected those values for *YA* and *OA* at which *MCoW* was minimal. All studies directly comparing *YA* and *OA* were selected for meta-analyses. From all studies reviewed, the average *GCoW* in *YA* was 3.4 ± 0.4 J/kg/m and 3.8 ± 0.4 J/kg/m in *OA* (~12% more in *OA*), and the average *NCoW* in *YA* was 2.4 ± 0.4 J/kg/m and 2.8 ± 0.5 J/kg/m in *OA* (~17% more in *OA*). Our meta-analyses indicated a statistically significant elevation of both *GCoW* and *NCoW* (p < 0.001) for *OA*. In terms of *GCoW*, *OA* expended about 0.3 J/kg/m more metabolic energy than *YA* and about 0.4 J/kg/m more metabolic energy than *YA* in terms of *NCoW*. Our study showed a statistically significant elevation in *MCoW* of *OA* over *YA*. However, from the literature it is unclear if this elevation is directly caused by age or due to an interaction between age and methodology. We recommend further research comparing *MCoW* in healthy *OA* and *YA* during “natural” over-ground walking and treadmill walking, after sufficient familiarization time.

## Introduction

## Glossary

- *WS* in [m/s]: the measured walking speed either over-ground or on a treadmill.
- $\dot{V}{O}_{2}$ in [ml/kg/min]: the rate of oxygen consumption.
- *EEq* of $\dot{V}{O}_{2}$ in [J/kg/min]: Energetic Equivalent of the oxygen consumed during walking.
- *RMR* in [J/kg/min]: the rate of metabolic energy consumption during quiet standing or sitting (Resting Metabolic Rate).
- *MCoW* in [J/kg/m]: metabolic energy consumed, per kilogram of body mass per meter travelled, typically expressed as gross or net values (Metabolic Cost of Walking).
- *GCoW* in [J/kg/m]: *EEq*/(*WS* ×60) (Gross Cost of Walking).
- *NCoW* in [J/kg/m]: (*EEq* – *RMR*)/(*WS* ×60) (Net Cost of Walking).
- *RER* [dimensioneless]: the amount of carbon dioxide produced divided by the amount of oxygen consumed (Respiratory Exchange Ratio).
- *OA*, *YA*: (Old Adults, mean age ≥ 59 years) and (Young Adults, mean age 18–41 years).

Walking is the most prevalent physical activity for humans and may account for up to thirty percent (30%) of an adult’s daily energy expenditure^1^. Hence, the Metabolic Cost of Walking (*MCoW*), defined as metabolic energy expended per meter travelled, is an important variable in daily life and has been studied extensively. It is generally presupposed that humans, under normal circumstances, select their gait parameters (like step frequency and walking velocity) to minimize *MCoW* e.g.^2–4^. *MCoW* has been studied in healthy children, teenagers, young and elderly adults, at preferred or fixed walking speeds and during both over-ground and treadmill walking. One major finding that has been reported by several studies is that *MCoW* is higher in healthy Older Adults e.g.^5–7^ (*OA*; to be consistent with the analyzed literature, defined here as mean age ≥ 59 years) than in Young Adults (*YA*; here defined as mean age from 18–41 years)_,_ although a recent extensive study has found contradictory results^8^. An elevated *MCoW* may cause *OA* to become fatigued more quickly and walk less, which in turn may lead to functional limitations and reduced societal participations. Currently, no quantitative analyses are present in the literature that directly compare the results across studies on *MCoW* in *YA* and *OA*, or on the differences between these two groups. Such analyses may be important, for example to decide whether the elevation in *MCoW* of *OA* over *YA* is clinically relevant.

Directly comparing the results in the literature across different studies on *MCoW* is not without problems. A first difficulty comparing quantitative measures across studies arises from the ways in which *MCoW* was expressed. Several studies expressed *MCoW* only in terms of Gross Cost of Walking (*GCoW*) e.g.^9,10^ which is the total amount of metabolic energy consumed per kilogram of body mass per meter travelled. Other studies expressed *MCoW* only in terms of Net Cost of Walking (*NCoW*) e.g.^5,7,11^ which is assumed to be the total amount of metabolic energy expended due to walking *per se* (e.g., due to cross-bridge cycling and the *Ca*^*2+*^ pump in the muscle) calculated by subtracting first the Resting Metabolic Rate (*RMR)* from Energetic Equivalent (*EEq*) of the oxygen consumed and then divided by the Walking Speed (*WS)*. *GCoW* and *NCoW* are different in nature and quantity, making it difficult to interpret results across studies that only reported either one of these. For example, an increase in *NCoW* does not necessarily mean an increase in *GCoW* (and vice-versa) as there is evidence in literature that *RMR* may be different between *YA* and *OA*^11,12^. In addition, some of these studies do not report *RMR* values making it impossible to convert *GCoW* to *NCoW* and vice versa. A second difficulty is that *MCoW* and (rate of) energy expenditure have been reported in various units such as ml of O_2_/kg/m^9^, J/kg/m^11^ or W/kg^13^ and it is not trivial to convert one to another. For example, to convert from ml of O_2_/kg/m to J/kg/m, Respiratory Exchange Ratio (*RER*) is required, which typically differs among subjects and is often not reported. A third problem arises from differences in walking speed at which *MCoW* was measured. For example, Stoquart *et al*.^14^ reported *MCoW* in *YA* at a walking speed of 1.1 m/s, whereas Davies and Dalsky^15^ reported *MCoW* in *OA* at a speed of 1.5 m/s. This is a problem because it is known that *MCoW* depends on walking speed e.g.^4,16,17^. Some studies used an identical fixed speed for *YA* and *OA*, but the values at which speed was fixed varied across studies, (e.g., 1.0 m/s in^5^ and 1.3 m/s in^18^). All of these difficulties mentioned above hamper the direct comparison of the results across different studies and therewith the observation of the actual values of *MCoW* in *YA* and *OA*.

Recently, Aboutorabi *et al*.^19^ performed a literature review on effects of aging on the temporo-spatial (gait) parameters, kinematics, kinetics and energetics of gait. They concluded that aging led to an increase in *MCoW*. However, their list of included studies was not exhaustive, they did not address the difficulties described above and, more importantly, they did not quantify the elevation identified.

The primary aims of the present study were to provide an overview of the literature on *MCoW* in *YA* and *OA* and to investigate quantitatively the elevation of *MCoW* related to aging on the basis of the published literature. To do so, we first carefully searched the literature on *MCoW* in both *YA* and *OA* and selected all relevant scientific articles using the Preferred Reporting Items for Systematic Review and Meta Analyses *(PRISMA)* method. We then statistically analyzed group differences, and then selected studies in which both *YA* and *OA* were measured in the same experimental setup. To estimate the effect size of *MCoW* between *YA* and *OA* we subsequently performed two meta-analyses, one on *GCoW* and the other on *NCoW* on these selected studies, that directly compared *YA* and *OA*.

## Methods

### Inclusion and exclusion criteria

We considered only studies published in peer reviewed journals that reported *MCoW* in healthy humans. In this study, we focused on healthy young and elderly adults and excluded all studies involving subjects with disabilities, comorbidities and a history of falling. We noted that the study of Malatesta *et al*.^6^ did not mention whether the value for *MCoW* was gross or net. Based on the reported values, we are fairly confident that they were *GCoW* values and have included the study in our analyses; the values for the two *OA* groups (G80 and G65) were averaged and used for our meta-analyses. We furthermore specifically comment on the exclusion of two studies from our analyses. First, Thomas *et al*.^20^ decided to pool the data of *MCoW* in their analysis as they did not find any significant difference in the Preferred Walking Speed (*PWS)* of healthy young and elderly women. Second, Pearce *et al*.^21^ also pooled *MCoW* for both *YA* and *OA* as they were only interested in the effect of walking surface on *MCoW*. The results of these studies are obviously unsuitable for our purpose of investigating an age-related elevation in *MCoW*.

### Search strategy

**Figure 1:**
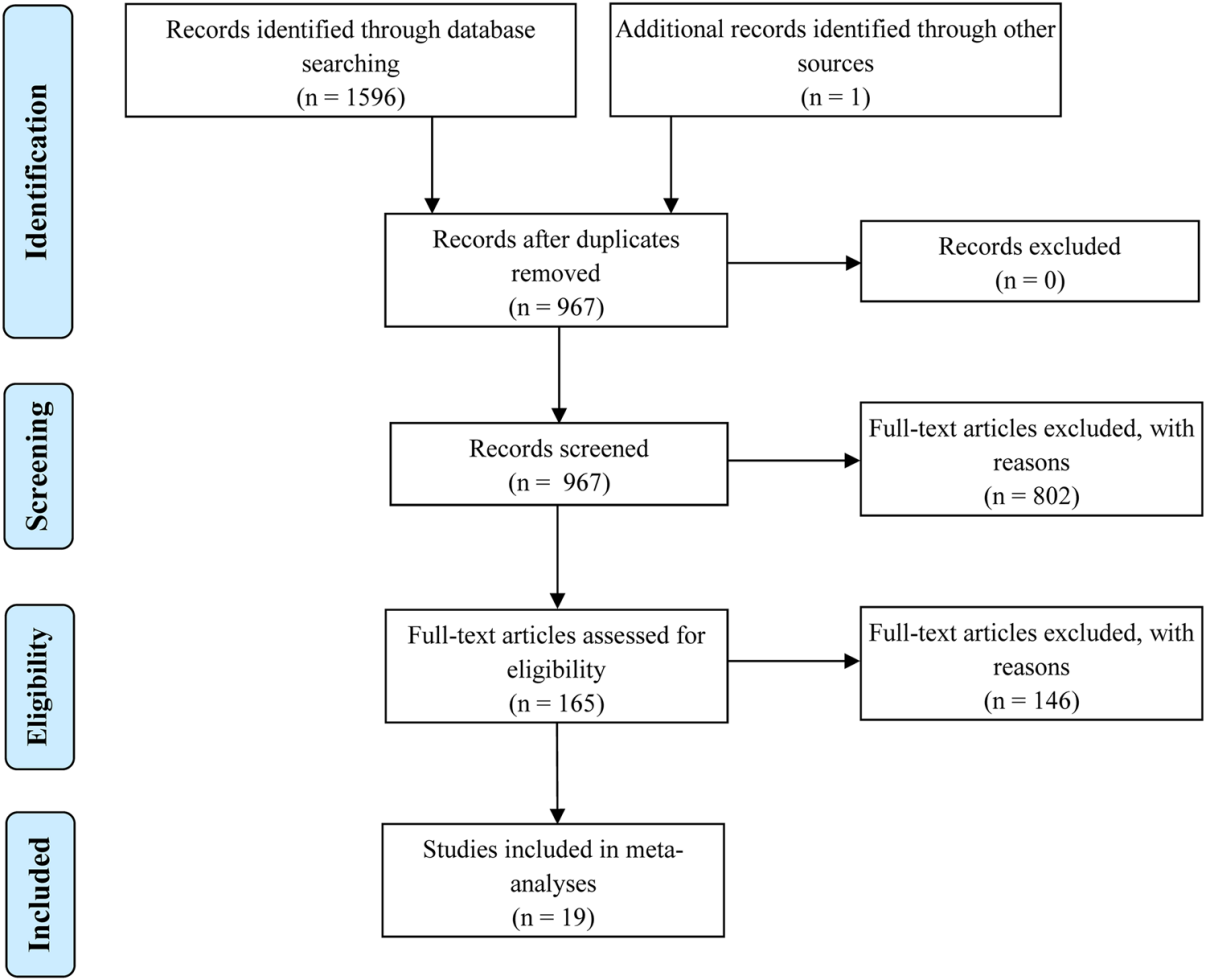
Flow-diagram of the search and selection strategy followed using *PRISMA* method.

A review protocol was developed based on the Preferred Reporting Items for Systematic Reviews and Meta-Analysis (*PRISMA*)-statement (see: www.prisma-statement.org)^22^. A comprehensive search was performed in the bibliographic databases PubMed, Embase.com, Ebsco/SPORTDiscus and Wiley/Cochrane Library in collaboration with a medical librarian. Databases were searched from inception up to 14 Aug 2018. The following terms were used (including synonyms and closely related words) as index terms or free-text words: “Aged”, “Elderly”, “Gait”, “Walking”, “Energy metabolism”. The search was performed without date, language or publication status restriction. The full search strategies for all databases can be found in the Supplementary Information (see: Appendix D). The initial list of the papers was selected through evaluation of the Title, Keywords and Abstract. Then, a further selection based on screening of the Methodology, Protocol and the Aims of the studies was conducted by all authors independently. A flow-diagram for *PRISMA* method is shown in Fig. 1. Screening was done simultaneously using a manual search process undertaken in EndNote (Clarivate Analytics) and online by the open-source web application Rayyan QCRI (Qatar Foundation & Qatar Computing Research Institute).

### Definition of age group

In our analyses we followed the definition of age group (*OA* vs *YA*) used by the authors of the individual studies. This resulted in a somewhat arbitrary overall definition of *OA* group of 59 years and older. To test for robustness against definition of the age group *OA*, we also performed statistical tests following the definition by the United Nations (*UN*) of elderly, being of the age of 65 and older, which showed only a marginal influence of the difference in definition of *OA*. The only study in the meta-analyses that had *OA* below 65 was that of Jones *et al*.^23^.

### Overview of analyses

From the selected studies, we extracted all data on *MCoW* and calculated, if not already reported, *GCoW* and *NCoW*. If the unit in which *MCoW* was reported was not in the *SI* unit of J/kg/m, we converted the reported values accordingly. Studies typically either report *MCoW* for self-selected or preferred walking speed. If studies reported *MCoW* measured at multiple speeds, we selected *MCoW* values for both *YA* and *OA* at the speeds at which *MCoW* values were minimal as it is known that under normal circumstances humans tend to self-select walking speeds in close proximity of the minimal *MCoW* speed. Using the selected data, we first reported *MCoW* in both *YA* and *OA* and statistically analyzed group differences in walking speed (since the selected studies included both the experimentally fixed and preferred walking speeds) and *MCoW* between *YA* and *OA*. We then performed two meta-analyses, one on *GCoW* and one on *NCoW*, on the selected studies that directly compared *YA* and *OA*. We did this in order to account for the heterogeneity across the studies and differences in the experimental sample size and to calculate the effect size of *MCoW* between *YA* and *OA*.

### Calculations of *MCoW*

Not all of the included studies reported all the variables of interest (being *GCoW, NCoW, RMR* and *RER*). If any of these variables was not reported, we attempted to calculate its mean and standard deviation from the other variables reported. In general, calculating means is straightforward, but calculating standard deviations is not possible unless the individual sample data are known, which was generally not the case. Whenever standard deviations were not given, we estimated them (see Appendix A for detailed overview of the calculated variables of interest and Discussion). In general, we used the following two equations to estimate standard deviations. For addition and subtraction of two quantities (say z = a + b or z = a − b), the estimated standard deviation of z (${{\rm{\sigma }}}_{{{\rm{z}}}_{{\rm{est}}}}$) was calculated as:

1 $${{\rm{\sigma }}}_{{{\rm{z}}}_{{\rm{est}}}}=\sqrt{{({{\rm{\sigma }}}_{a})}^{2}+{({{\rm{\sigma }}}_{b})}^{2}}$$

with ${{\rm{\sigma }}}_{a}$ and ${{\rm{\sigma }}}_{b}$ the known standard deviations of a and b. For multiplication and division of two quantities (say z = ab or z = a/b), the estimated standard deviation was calculated as:

2 $${{\rm{\sigma }}}_{{{\rm{z}}}_{{\rm{est}}}}=z\sqrt{{(\frac{{{\rm{\sigma }}}_{a}}{a})}^{2}+{(\frac{{{\rm{\sigma }}}_{b}}{b})}^{2}}$$

Finally, we note here that in some studies *RMR* was measured while sitting, whereas in other studies it was measured while standing. *RMR* is close to 14% lower during sitting than during standing^24^ and this may influence the values obtained for either *GCoW* or *NCoW* when only one of these was reported.

### Statistical analysis

We checked for normality of the data using a Shapiro-Wilk hypothesis test along with violinplots and QQ plots. The result of the test and the visual inspection of the plots showed that the data was non-normally distributed. Additionally, Levene’s test was used to check for the equality of variances and no violation of the equality was observed in the data. Based on these outcomes we chose to use a non-parametric Wilcoxon rank sum test.

We analyzed whether walking speeds at which *MCoW* (for both *GCoW* and *NCoW)* was measured differed between *YA* and *OA* using an independent two-sided non-parametric Wilcoxon rank sum test. After that, we used an independent one-sided non-parametric Wilcoxon rank sum test to test for an elevation of *GCoW* and *NCoW* of *OA* over *YA*. The open-source software JASP (version 0.9.0.1) was used for these statistical tests. However, from these analyses it is not possible to draw a definitive conclusion on the effect size of *GCoW* and *NCoW* between *YA* and *OA*. This is because not all selected studies directly compared *YA* and *OA*, and across the selected studies there were considerable differences in sample size, experimental methodology and various other factors like equipment, ethnicity and gender of subjects, etc. To be able to calculate the (pooled) effect size of *MCoW* between *YA* and *OA*, we selected only those studies that directly compared *YA* and *OA*, 19 in total (see Fig. 1) and performed meta-analyses on them. We ended up with 13 studies for *GCoW* and 18 studies for *NCoW* (both being a large-scale meta-analysis^25^), because in 5 studies *RMR* was not mentioned and only *GCoW* or *NCoW* was reported. If some studies did not report *RMR* it became impossible to calculate *GCoW*/*NCoW* when only one was reported. Some of these studies measured *MCoW* at the same fixed walking speed in *YA* and *OA*, while others measured *MCoW* at individually preferred walking speeds, that differed between *YA* and *OA*.

To account for the possible heterogeneity across studies, we used a continuous random-effects model. The studies were weighed according to their respective sample sizes and standard deviations of their group mean values. The meta-analyses were performed using the open-source software packages Open Meta[Analyst] (Brown University of Public Health, Providence, USA) and JAMOVI (version 0.9.1.12). The two-arm metric of mean differences of individual studies was used to calculate the pooled effect size, the 95% confidence intervals and the 95% prediction intervals. Pooled mean differences were calculated as *MCoW* for *OA* minus *MCoW* for *YA* for both *GCoW* and *NCoW*. In addition, we calculated for both *GCoW* and *NCoW* the pooled standardized mean difference, which is the (pooled) mean difference normalized relative to pooled standard deviation computed using Hedge’s G.

### Validation and sensitivity tests

To assess the validity of the a-priori assumption of heterogeneity in the meta-analyses, we used the three standard tests of heterogeneity (Cochran’s Q, tau^2^ and I^2^). A sensitivity analysis was performed on each of the studies to check for the robustness of the meta-analysis using the Leave-One-Out method.

### Risk of bias assessment

The appraisal tool for cross-sectional studies (AXIS^26^) was used as a tool to conduct the risk of bias assessment for the individual studies included in our meta-analyses. In short, all the studies were judged systematically on each of the specific sections of the articles starting from Introduction, Methods, Results to Discussion and other relevant parameters. Twenty specific questions were used to evaluate each of these sections of the articles with the option of answering them by a ‘YES’, ‘NO’ or ‘DO NOT KNOW’. Additionally there is an option to leave specific comments explaining the answers provided for each of the questions.

## Results

**Figure 2:**
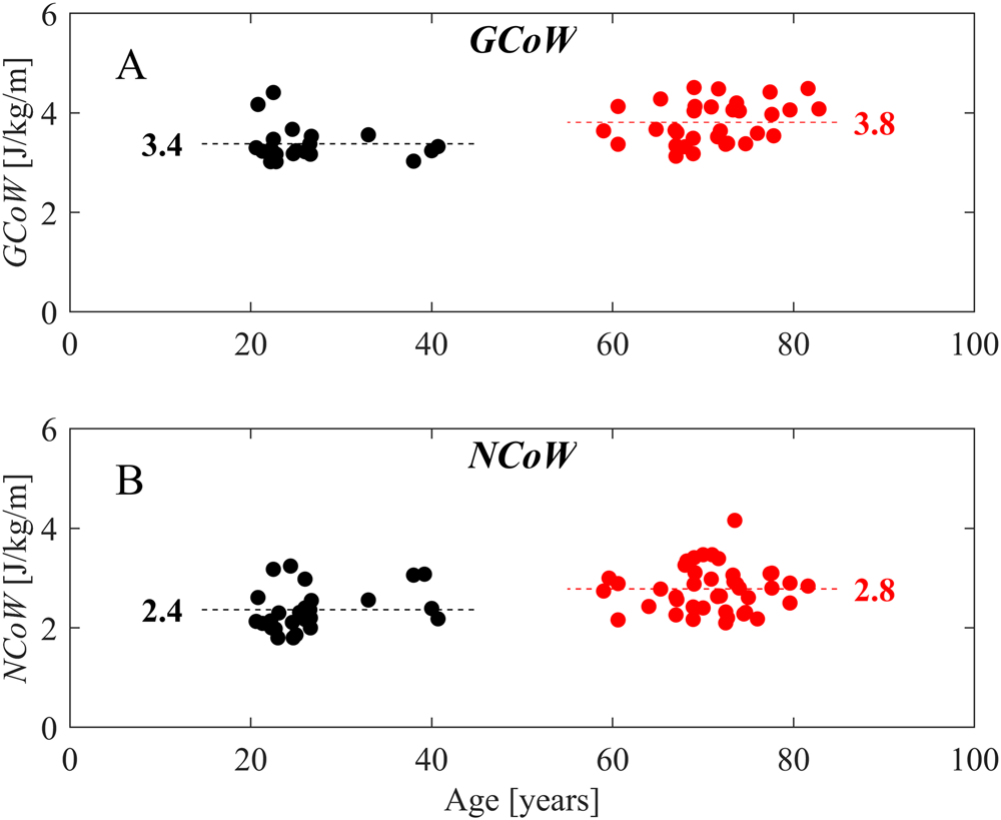
Metabolic Cost of Walking across the mean age in each study. (**A**) Gross Cost of Walking across age. (**B**) Net Cost of Walking across age. Black solid circles represent *YA* and the red solid circles *OA*. Each of the circles is a mean* GCoW* value or a mean *NCoW* value from a selected study. The dashed straight lines (black and red) show the mean metabolic cost of a particular age-group for both *GCoW* and *NCoW*^40–56^.

Figure 2A,B depict the mean *GCoW* and *NCoW* values as function of mean age of a group of studies reporting values for *GCoW* and/or *NCoW*. The data were then grouped into either *YA* (black dots) or *OA* (red dots) as was done in the selected studies. The mean walking speeds were found to be not statistically different between these groups (1.2 ± 0.2 m/s in *YA* vs 1.2 ± 0.2 m/s in *OA*, mean ± SD, p = 0.58). It was found that the average *GCoW* was 12% higher in *OA* (3.8 ± 0.4 J/kg/m) than in *YA* (3.4 ± 0.4 J/kg/m) and that this elevation was statistically significant (p < 0.001). The average *NCoW* was 17% higher in *OA* (2.8 ± 0.5 J/kg/m) than in *YA* (2.4 ± 0.4 J/kg/m); this elevation was also statistically significant (p < 0.001). These results indicate an elevation in *GCoW* and *NCoW* in *OA* compared to *YA*.

**Figure 3:**
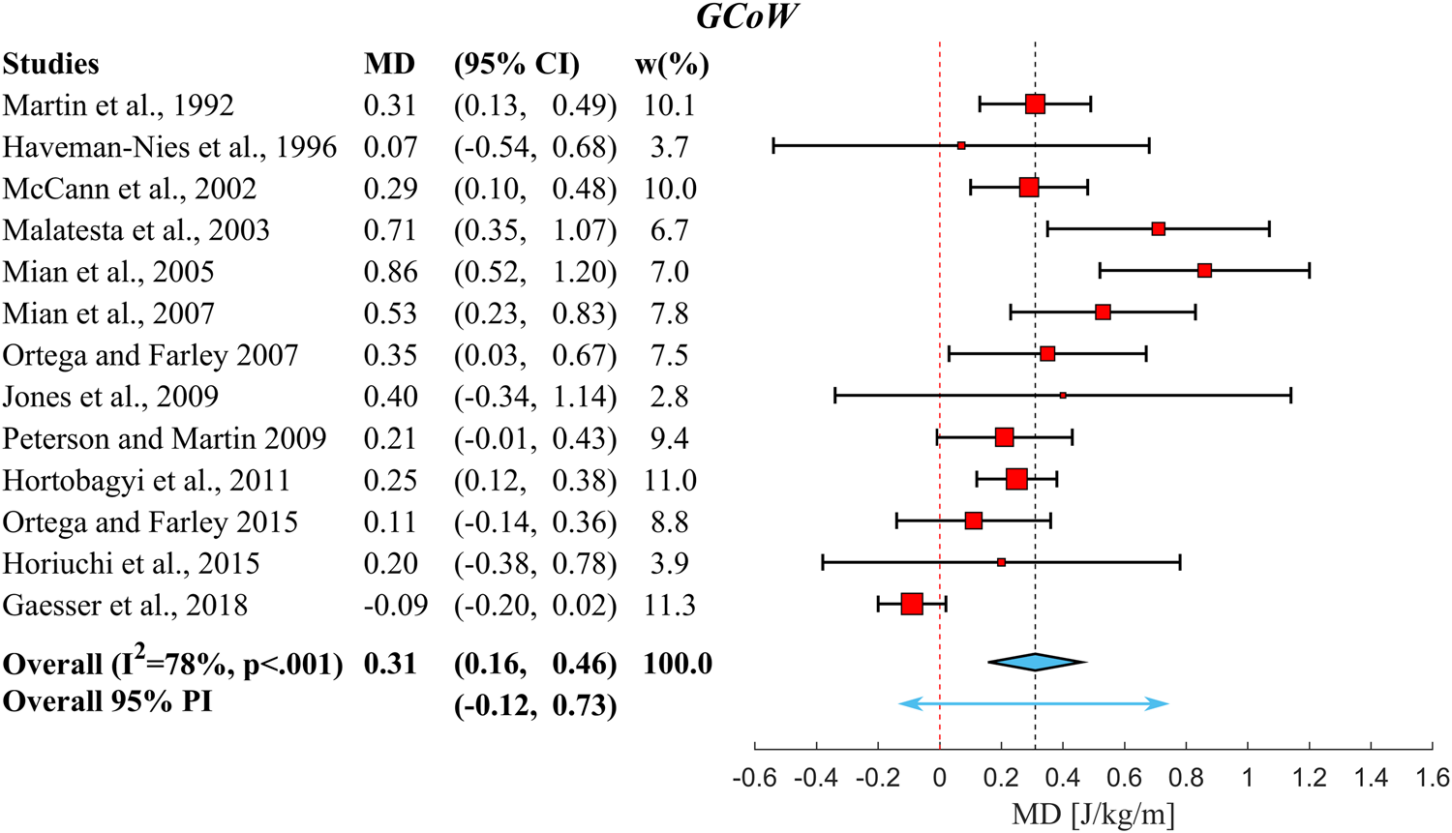
Overview of the results of the meta-analysis using a random effects model on the differences in Gross Metabolic Cost of Walking between *YA* and *OA*. A positive Mean Difference (*MD*) means that *OA* have higher *GCoW* than *YA*. The position of the red squares corresponds to the *MD* value per study and the horizontal black line to the 95% *CI*. The size of the square is proportional to the relative weight of that study w(%) to compute the overall *MD* (blue diamond and vertical black dashed line). The width of the diamond represents the 95% *CI* of the overall *MD* and the blue arrow represents the 95% *PI* of the overall *MD*. If a 95% *CI* spans zero (indicated by the red vertical dashed line), that study has found no significant elevation in *GCoW*. This meta-analysis shows a significant overall increase in *GCoW* of *OA* over *YA* (p < 0.001). Note that the p-value mentioned in the forest plot is the p-value for the Cochran’s Q heterogeneity test.

**Figure 4:**
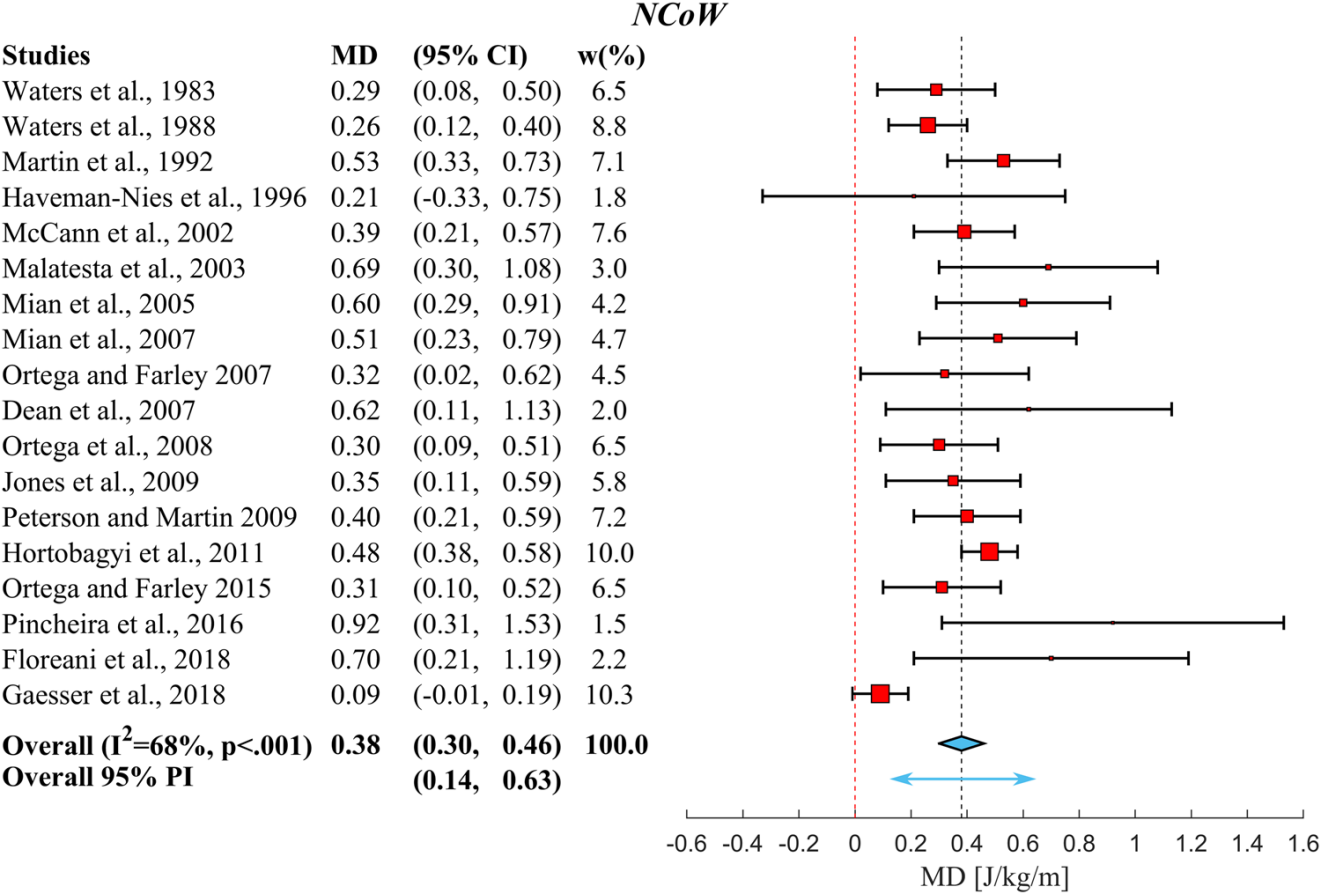
Overview of the results of the meta-analysis using a random effects model on the differences in Net Metabolic Cost of Walking between *YA* and *OA*. Symbols and conventions are the same as in Fig. 3. This meta-analysis shows a significant overall increase in *NCoW* of *OA* over *YA* (p < 0.001). Note again that the p-value mentioned in the forest plot is the p-value for Cochran’s Q heterogeneity test.

After this, a selection of studies was made from the studies in Fig. 2, which directly compared *YA* and *OA*. No statistically significant differences (p = 0.59) were found in the overall walking speeds between *YA* (1.2 ± 0.2 m/s) and *OA* (1.2 ± 0.1 m/s) of these studies. We then used these studies to perform two meta-analyses to calculate mean difference in *MCoW* between *OA* and *YA*. Figures 3 and 4 depict forest plots for *GCoW* and *NCoW*, respectively, showing for each of the individual studies the mean difference (red squares) and 95% Confidence Interval (95% *CI*, black lines), as well as the estimated pooled mean difference (center of blue diamond and dashed black summary line), 95% *CI* (width of blue diamond) and 95% Prediction Interval (*PI*, blue arrow). The size of the relative weight (*w*) of a particular study (indicated by the size of the squares in Figs 3 and 4) was dependent on the study sample size and standard deviation of the study sample mean (see^27^). Both for *GCoW* and *NCoW* we found a pooled mean difference that was statistically significant (0.31 J/kg/m and 0.38 J/kg/m, respectively, both p < 0.001). Note that the pooled mean difference of *NCoW* was 23% higher than that of *GCoW*; this indicates a difference in *RMR* between *YA* and *OA*, which has been reported before in the literature^11,12^. The 95% *CI* estimates the uncertainty of capturing the mean difference. By taking into account the heterogeneity across studies, the 95% *PI* estimates the interval in which an effect size from a future study assessing the same outcome will lie^28^.

In order to assess the magnitude of the (pooled) effect size, we calculated the pooled *standardized* mean difference (Hedge’s G), which was 0.65 for *GCoW* and 1.00 for *NCoW*. Quantitatively, according to general guidelines^29^, the effect size was in the range of medium to large for *GCoW* and large for *NCoW*.

### Validation and sensitivity tests

In order to assess the validity of our *a-priori* assumption of heterogeneity, we performed three standard statistical tests for heterogeneity. Firstly, we computed Cochran’s Q, which is the weighted sum of squared differences between the individual study effects and the pooled effect across studies. Cochran’s Q was 55.7 (p < 0.001) and 53.2 (p < 0.001) for *GCoW* and *NCoW*, respectively, indicating a statistically significant heterogeneity. Since Cochran’s Q test has limited power to detect heterogeneity when using a small number of studies^30^, we secondly calculated I^2^, which is a measure of variation in the pooled effect size proportional to the total amount of variance and is independent of the number of studies. The calculated values of I^2^ were 78% and 68% for respectively *GCoW* and *NCoW* (0% meaning complete homogeneity). Even though there are no known concrete thresholds of I^2^, these values indicate substantial heterogeneity (see^30^). Thirdly, we calculated tau^2^ values, which show the variance among the studies; these were 0.043 (with Standard error = 0.028) for *GCoW* and 0.013 (with Standard error = 0.01) for *NCoW* (a tau^2^ value of greater than one indicates a meta-analysis should not be performed on the selected studies). The non-zero values of tau^2^ again indicate that there was heterogeneity across studies. The overall results support our choice of using a random effects model for our meta-analyses.

**Figure 5:**
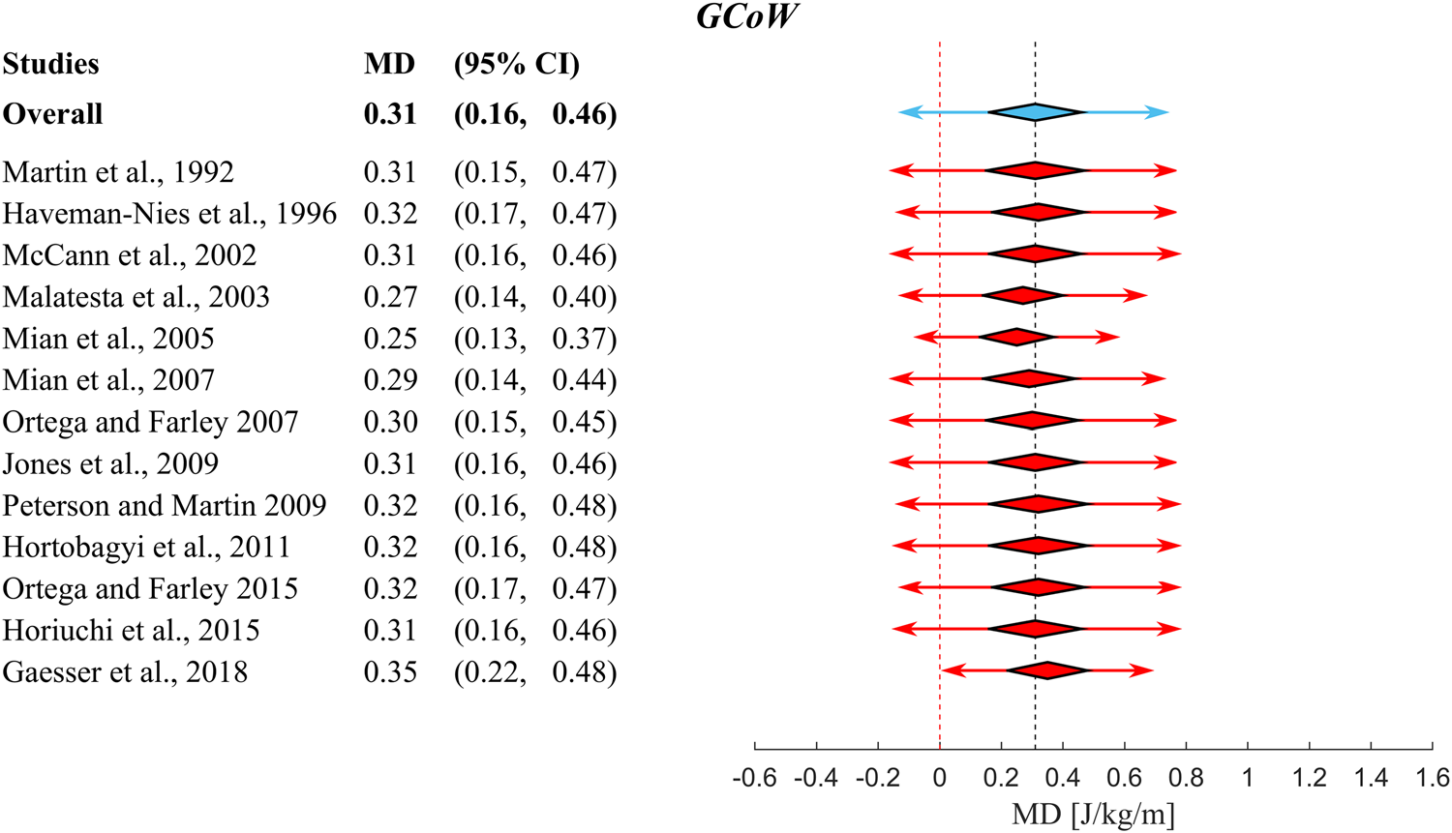
Leave-one-out plot of the meta-analysis on *GCoW*. The blue diamond now represents the pooled *MD* with its 95% *CI*, and the red diamonds represent the pooled *MD* along with its 95% *CI* when leaving out the study indicated. The arrows represent the 95% *PI* for the overall pooled *MD*. This figure shows that leaving out any of the studies does not cause the 95% *CI* to span zero for any of the red diamonds. Thus, the statistical significance of the elevation of *GCoW* in *OA* is not affected by leaving out any one of the studies.

**Figure 6:**
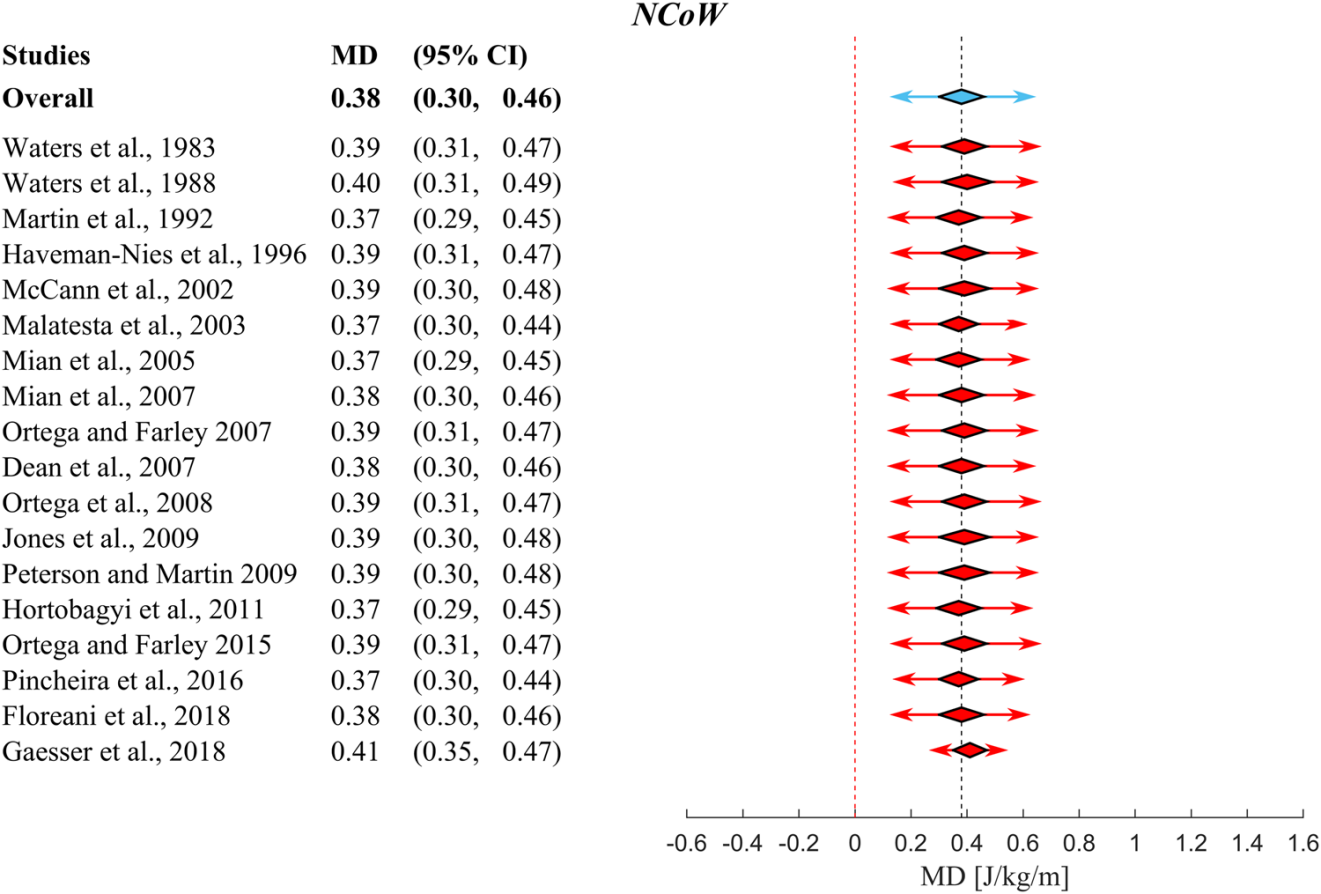
Leave-one-out plot of the meta-analysis on *NCoW*. Symbols and conventions are the same as in Fig. 5. As with *GCoW*, the statistical significance of the elevation of *NCoW* in *OA* is not affected by leaving out any one of the studies.

Finally, we performed sensitivity analyses of the meta-analyses using the Leave-One-Out method, which involves repeating the meta-analyses leaving out one of the studies in turn (see Figs 5 and 6). Each red diamond in Figs 5 and 6 represents the estimated pooled mean difference (middle of diamond) and 95% *CI* (the width of the diamond) while leaving out the indicated study from the meta-analyses. The red arrow indicates the 95% *PI*. These figures show that leaving out any one of the studies would not have affected the statistical significance of the overall results of the meta-analyses (p < 0.001 for both *GCoW* and *NCoW*). These figures furthermore show that the choice of definition of *OA* group did not influence our conclusions; by leaving out^23^, the only study which defined *OA* to be below 65 years old (being >59), pooled *MD* only marginally changed. Note, however, that the 95% *PI* for leaving out^8^ in the meta-analysis for *GCoW* does change; the interval does not span zero. This is because this study found a higher mean value for *YA* than *OA*.

### Risk of bias assessment

The risk of bias assessment was done using the AXIS tool as mentioned in the Methods section. To summarize, none of the studies reported an a-priori statistical power calculation and few of the studies had a possible selection bias in recruiting human subjects for the experiments. Overall, the studies were found to be of good quality. For a detailed description of the assessment please see Appendix B.

## Discussion

We set out to report and pictorially represent *MCoW* in healthy *YA* and *OA* and to investigate the quantitative effect of age on *MCoW* through a literature review and meta-analyses. We extracted all data on *MCoW* in healthy *YA* and healthy *OA* and calculated, if not already reported, *GCoW* and *NCoW* in units of J/kg/m. If studies reported *MCoW* measured at multiple speeds, we selected the speed and *MCoW* values for *YA* and *OA* at which *MCoW* was minimal. An analysis of the compilation of *GCoW* and *NCoW* values from the literature as a function of age (see Fig. 2) showed a statistically significant elevation for *OA* over *YA*. To calculate the pooled effect size of *MCoW* between *YA* and *OA*, we performed two meta-analyses on the selected studies that directly compared *YA* and *OA*. In terms of *GCoW*, *OA* expended about 0.31 J/kg/m more than *YA* at similar walking speeds (walking speeds included in the meta-analyses were not significantly different between *OA* and *YA*). In terms of *NCoW*, *OA* expended about 0.38 J/kg/m more than *YA*. There was (~23%) more elevation for *NCoW* than *GCoW* for *OA* over *YA*. These results were found to be robust against choices made in statistical models and parameters. Figures 5 and 6 furthermore showed that leaving out any of the studies would not have affected the statistical significance of the overall results of the meta-analyses (in terms of pooled *MD* and 95% *CI*). These figures also show that the choice of definition of *OA* group did not influence our conclusions; even leaving out^23^, the only study in the meta-analyses that had *OA* below 65 years of age, only marginally changed the results. Although it is difficult to directly assess the clinical relevance from statistical results, the standardized effect sizes were medium to large, indicating that these differences are indeed clinically relevant^31^.

For our analyses, we have expressed *MCoW* in terms of both *GCoW* and *NCoW* and in units of J/kg/m. Since not all studies used in our analyses reported the variables of interest, or reported them in different units, we attempted to determine these from the other variables that were reported. In general, as stated in the *Methods*, calculating means is straightforward. However, calculating standard deviations is not possible unless the individual sample data are known, which were typically not reported. Since standard deviations are required to perform statistical tests we had to resort to estimating them. We estimated standard deviations conservatively (see: *Calculations of MCoW* in *Methods* and in Appendix A), which decreased the likelihood of finding a statistically significant elevation. Nevertheless, our meta-analyses showed a significant overall elevation in both *GCoW* and *NCoW* of *OA* over *YA* and thus our results were not critically influenced by the estimation of the standard deviations.

In our analyses, we combined studies that measured *MCoW* at different walking speeds (e.g. at a range of fixed values versus self-selected/preferred walking speed). As stated before, when several fixed speeds were used in a particular study, we selected the speed accompanied by the lowest *MCoW*. Typically, *PWS* is measured in advance of the actual experiment and this speed is then also fixed during the experiment itself. Importantly, before conducting the analyses on *MCoW* we analyzed group differences in walking speed and showed that the preferred walking speeds were not statistically significantly different, and in fact were very similar. All in all, we can say that it was safe to combine ‘self-paced’ and ‘fixed-speed’ conditions studies.

A general concern with meta-analyses is the risk of positive publication bias^32^, also known as the ‘file-drawer’ phenomenon. The general way of assessing a positive publication bias, is to make use of a Funnel plot and to test for asymmetry. In a Funnel plot, standard error is shown against mean difference (effect size). Based on funnel plots, there is no reason to assume the presence of positive publication bias for *GCoW* (see: Appendix C for details). For *NCoW* this is less clear as we found a statistically significant asymmetry in the Funnel plot, which can be caused by publication bias. However, the majority of the papers in the Funnel plot for *NCoW* are also in the Funnel plot for *GCoW*. It is therefore less likely, albeit not impossible, that a positive selection bias is present for *NCoW* only. In addition, several other factors also introduce asymmetry (e.g. heterogeneity within and among the selected studies and differences in methodologies of studies performed^33^). Unfortunately, there is no decisive way to test for the presence of positive publication bias.

Having established that there is a difference in *MCoW* between *YA* and *OA*, a question arises whether the elevated *MCoW* in healthy *OA* is a direct effect of age, or is due to age-related confounders? There is indeed some evidence in the literature that points to a possible effect of age on *MCoW*. Malatesta *et al*.^6^ measured *MCoW* in ten *YA* and two groups of *OA* (ten subjects from 60–69 years old and ten subjects from 77–86 years old) and found that for all the five speeds measured, significant differences in *MCoW* were found between *YA* and the oldest *OA* group, and only for the two highest speeds between *YA* and youngest *OA* group. In addition, in a large study, Gaesser *et al*.^8^ measured *MCoW* in 96 healthy *YA* and in 94 healthy *OA* that were divided into three age sub-groups: 31 subjects from 60–64 years old, 34 from 65–69 years old and 29 from >69 years old. As stated before, they found no statistically significant differences in *GCoW* between *YA* and any of *OA* groups (actually, the mean *GCoW* in *YA* was higher). However, for *NCoW* they reported a significantly elevated cost only for the oldest group (>69 years old, p = 0.04). Be that as it may, such an age related influence does not explain the contradictory results of Gaesser *et al*.^8^ with our meta-analyses. Since we have grouped age ≥59 as *OA*, our mean difference should be, if anything, lower than the mean difference between *YA* and the oldest *OA* group from^8^, which is clearly not the case. Thus, based on the literature, it cannot be determined whether the elevation found in our study is directly caused by age, or whether age-related confounders play a role.

A possible confounder is that *OA* need more familiarization time than *YA* in experimental settings. For example, it has been shown that *OA* need substantially more time than *YA* to find their natural gait on a treadmill^34^. Wass *et al*.^35^ showed that *OA* require at least 15 minutes of familiarization time on a treadmill to approximate natural over-ground gait kinematics for a single walking speed at level grade of treadmill. Most of the studies in our meta-analyses were conducted on a treadmill and, unfortunately, often did not provide details about the familiarization time, rendering a correlational analysis between familiarization time and *MCoW* impossible. Based on reported total familiarization time across conditions, the average familiarization time *per walking condition* ranged from 1.2 minutes e.g.^36^ to 10 minutes e.g.^12^ which may not have been enough to arrive at normal gait and *MCoW*^34^. Gaesser *et al*.^8^ performed their study on a treadmill at a fixed speed (1.34 m/s) and did not find an elevation of *MCoW* in *OA* over *YA*. Interestingly, in that study though no details on the familiarization protocol were given in the article, but ample familiarization time was provided (personal communication). To conclude, at this point it is unclear if treadmill walking in combination with insufficient familiarization time is a confounding factor in the elevation in *MCoW* in *OA*.

If insufficient familiarization time in treadmill experiments is the true reason for elevation in *MCoW* of *OA* over *YA*, one would expect that the elevation in *MCoW* would not be present when measured during normal over-ground walking. In the two studies in our meta-analyses that measured *MCoW* during over-ground walking^37,38^, *MCoW* was elevated in *OA* over *YA*, just as in the treadmill studies; (first two studies listed in Fig. 4). However, the preferred walking speed of *OA* group was significantly lower than that of *YA* group, which, as suggested by the authors, was the likely cause of the found elevation in *MCoW* of *OA* over *YA*.

Other possible confounders are differences in fitness level, activity level and anthropometrics. It is quite possible that the normal daily physical activity level differs between *YA* and *OA* and that this may influence the elevation in *MCoW* found in *OA*. Unfortunately, details about the daily physical activity level of participants are often not reported; typically the articles included in the meta-analyses report only that their subjects are all “fit and healthy” for both *YA* and *OA*. In addition, the body mass and body composition may also confound the results. Some of the studies in our meta-analyses statistically analyzed the differences in body mass and Body Mass Index (*BMI*), and found statistical differences between *YA* and *OA*. In our analyses, we used reported mass-normalized values of *MCoW* for both *YA* and *OA*, assuming that all participants have the same proportion of muscle mass to body mass, which may thus well not be the case and may influence the found results (see^39^) of our study. Since the studies analyzed here did not report individual data, we were not able to control for the effect of body composition on *MCoW* using an Analysis of Covariance (*ANCOVA*)^39^.

To conclude, our analyses show a robust and statistically significant elevation in *MCoW* of *OA* over *YA* when walking at comparable speeds. However, it cannot be determined on the basis of the literature whether this elevation is caused directly by age or is caused by age-related confounders. This merits further research comparing *MCoW* in healthy, active and anthropometrically matched *OA* and *YA* during both “natural” over-ground walking and treadmill walking at fixed walking speeds with sufficient familiarization time.

## Supplementary information

Appendices

## Data Availability

The datasets generated during and/or analyzed during the current study are available from the corresponding author on reasonable request.
