# Supplementary material for: The Metabolic Cost of Walking in healthy young and older adults – A Systematic Review and Meta Analysis
Source: Sci Rep. 2019 Jul 10;9:9956. doi: 10.1038/s41598-019-45602-4 (PMC6620279; doi:10.1038/s41598-019-45602-4)
Supplement: Supplementary file 1 — Appendices [file 41598_2019_45602_MOESM1_ESM.pdf]

Supplementary information

The Metabolic Cost of Walking in healthy young and older adults – A Systematic Review and Meta Analysis

Sauvik Das Gupta[1,2], Maarten F. Bobbert[1] and Dinant A. Kistemaker[1,a]

[1] Department of Human Movement Sciences, Faculty of Behavioural and Movement Sciences
Vrije Universiteit Amsterdam, Amsterdam Movement Sciences, The Netherlands

[2] Human Movement Biomechanics Research Group, Department of Movement Sciences, Biomedical Sciences Group, KU Leuven, Belgium

[a]Corresponding Author's Email: dinant.kistemaker@gmail.com

Appendix A - Calculations of *MCoW*

Calculation of MCoW from oxygen consumption

For those studies that did not report *MCoW*, but did report the oxygen consumption rate and *RER*, we used the Lusk Equation (Lusk, *J. Biol. Chem.* 1924) to calculate the mean Energetic Equivalent (*EEq*) of the oxygen consumed in units of J/kg/min:

$$EEq = (15962 + 5155 \cdot RER) \cdot (\dot{V}O_2 / 1000) \qquad (1)$$

The estimated standard deviation of *EEq* was calculated by:

$$\tilde{\sigma}_{EEq} = EEq\sqrt{\left(\frac{\sigma_{\dot{V}O_2}}{\dot{V}O_2}\right)^2 + \left(\frac{\sigma_{RER}}{RER}\right)^2} \qquad (2)$$

with $\dot{V}O_2$ and $\sigma_{\dot{V}O_2}$ the reported mean and standard deviation of oxygen consumption rate respectively, and *RER* and σ_{RER} the reported mean and standard deviation of *RER* respectively.

If *RMR* was not reported, but resting $\dot{V}O_2$ ($\dot{V}O_{2\_rest}$) was reported, *RMR* was calculated using the Lusk Equation:

$$RMR = (15962 + 5155 \cdot RER_{rest}) \cdot (\dot{V}O_{2\_rest} / 1000) \qquad (3)$$

The standard deviation of *RMR* was calculated using

$$\tilde{\sigma}_{RMR} = RMR\sqrt{\left(\frac{\sigma_{RER_{rest}}}{RER_{rest}}\right)^2 + \left(\frac{\sigma_{\dot{V}O_{2\_rest}}}{\dot{V}O_{2\_rest}}\right)^2} \qquad (4)$$

For the studies that reported the oxygen consumption rate but not *RER*, we estimated the mean *RER* to be equal to the average of the mean *RER*s from all the other studies that did report it. In that case we estimated the standard deviation of *RER* ($\tilde{\sigma}_{RER}$) using:

$$\tilde{\sigma}_{RER} = \sqrt{\sum_{i=1}^{n} \sigma_{RER_i}{}^2} \text{ and } \tilde{\sigma}_{RER\_rest} = \sqrt{\sum_{i=1}^{n} \sigma_{RER_{rest_i}}{}^2} \qquad (5)$$

for all *n* studies that reported *RER*.

Then, we calculated *GCoW* in units of J/kg/m using:

$$GCoW = \frac{EEq}{WS \times 60} \qquad (6a)$$

and, similarly *NCoW* using :

$$NCoW = \frac{EEq - RMR}{WS \times 60} \qquad (6b)$$

The estimated standard deviation of *GCoW* was calculated by:

$$\tilde{\sigma}_{GCoW} = GCoW\sqrt{\left(\frac{\tilde{\sigma}_{EEq}}{EEq}\right)^2 + \left(\frac{\sigma_{WS}}{WS}\right)^2} \qquad (7a)$$

The estimated standard deviation of *NCoW* was then calculated by:

$$\tilde{\sigma}_{NCoW} = \sqrt{\tilde{\sigma}_{GCoW}{}^2 + \sigma_{RMC}{}^2} \quad (7b)$$

when $\dot{V}O_2$ was measured at a fixed speed, σ_{RMC} was simply:

$$\sigma_{RMC} = \frac{\sigma_{RMR}}{WS \times 60}$$

If speed differed among subjects, then the estimation of σ_{RMC} was more involved:

$$\sigma_{RMC} = \frac{RMR}{WS \times 60}\sqrt{\left(\frac{\sigma_{RMR}}{RMR}\right)^2 + \left(\frac{\sigma_{WS}}{WS}\right)^2}$$

Calculation of GCoW/NCoW

For those studies that did report *MCoW*, but only reported either *GCoW* or *NCoW* (and *RMR*), *NCoW* or *GCoW* was calculated. *GCoW* was calculated using:

$$GCoW = NCoW + \frac{RMR}{WS \times 60} \quad (8a)$$

NCoW was calculated using:

$$NCoW = GCoW - \frac{RMR}{WS \times 60} \quad (8b)$$

In case *RMR* was not reported, only the reported values of *GCoW* and *NCoW* were used, since *RMR* is needed to calculate one from the other. The standard deviation of either *GCoW* or *NCoW* was estimated by:

$$\tilde{\sigma}_{GCoW} = \sqrt{\sigma_{NCoW}{}^2 + \sigma_{RMC}{}^2} \quad (9a)$$

$$\tilde{\sigma}_{NCoW} = \sqrt{\sigma_{GCoW}{}^2 + \sigma_{RMC}{}^2} \quad (9b)$$

Appendix B - Risk of Bias Assessment

The risk of bias assessment (table B1) is shown for all the studies included in the two meta-analysis on *GCoW* and *NCoW*. Specific comments about all or particular studies are mentioned in the rightmost column of the table.

Table B1 Risk of Bias Assessment

Serial Number	Question	Yes	No	Don't know/Comment
Introduction				
1	Were the aims/objectives of the study clear?	YES		
Methods				
2	Was the study design appropriate for the stated aim(s)?	YES		
3	Was the sample size justified?		NO	No details about *a-priori* power analysis or Bayesian statistical analysis were mentioned
4	Was the target/reference population clearly defined? (Is it clear who the research was about?)	YES		
5	Was the sample frame taken from an appropriate population base so that it closely represented the target/reference population under investigation?	YES		
6	Was the selection process likely to select subjects/participants that were representative of the target/reference population under investigation?	Mostly YES		For some studies it is difficult to say as it seems convenience sampling might have been done to recruit participants
7	Were measures undertaken to address and categorise non-responders?		NO	
8	Were the risk factors and outcome variables measured appropriate to the aims of the study?	YES		
9	Were the risk factors and outcome variables measured correctly using instruments/measurements that had been trialled/piloted or published previously?	YES		
10	Is it clear what was used to determine statistical significance and/or precision estimates? (e.g., p-values, confidence intervals)		NO	Confidence intervals mostly not considered. Only Pincheira et al.[36] and Waters et al.[38] reports it.<br>Mian et al. [7], Martin et al.[40] & Horiuchi et al.[9] say p=0.05 to determine statistical significance, whereas it should be p<0.05<br>Waters et al.[37] does not mention the criteria for statistical significance in the Methodology section<br>McCann & Adams [55] does not report the p or alpha value to determine statistical significance under statistical analyses under Methods section
11	Were the methods (including statistical methods) sufficiently described to enable them to be repeated?	YES		
Results				
12	Were the basic data adequately described?	YES		
13	Does the response rate raise concerns about non-response bias?		NO	Non-response bias not handled by the studies
14	If appropriate, was information about non-responders described?		NO	
15	Were the results internally consistent?		NO	[37] – Sample size in *OA* group not consistent in Table 1
16	Were the results presented for all the analyses described in the methods?	YES		
Discussion				

17	Were the authors' discussions and conclusions justified by the results?	YES		
18	Were the limitations of the study discussed?	YES		
Other				
19	Were there any funding sources or conflicts of interest that may affect the authors' interpretation of the results?		NO	
20	Was ethical approval or consent of participants attained?		NO	No mention of ethical approval or informed consent in [37] and [38].

Appendix C - Funnel Plots

For potential publication bias assessment, funnel plots were plotted for the meta-analyses on *GCoW* and *NCoW* (Figure C1). These plots are simple scatterplots of the mean differences of the included studies in the horizontal axis against the standard errors of the same studies in the vertical axis. A regression test for funnel plot asymmetry was also carried out because the pooled effect sizes for our meta-analyses were computed as mean differences (see: https://handbook-5-1.cochrane.org/chapter_10/10_4_3_1_recommendations_on_testing_for_funnel_plot_asymmetry.htm). In the absence of publication bias and heterogeneity, most of the studies will fall in the region within the "pseudo" 95% *CI* of the funnel, with an expected symmetric distribution of the studies about the vertical straight black line of *MD* (see Figure C1). Additionally we also note the results of the regression test for funnel plot asymmetry. For the meta-analysis on *GCoW*, the p-value of the test was statistically insignificant at an alpha level of 0.05 (p=0.250). However for the meta-analysis on *NCoW*, the p-value of the test is statistically significant at an alpha level of 0.05 (p=0.006), suggesting an asymmetry in the funnel plot. Funnel plot asymmetry can arise from many potential sources like publication bias, differences in methodologies of studies performed, heterogeneity across and/or within studies. Currently it is not possible to disentangle the reasons for the asymmetry in the plot for *NCoW*.

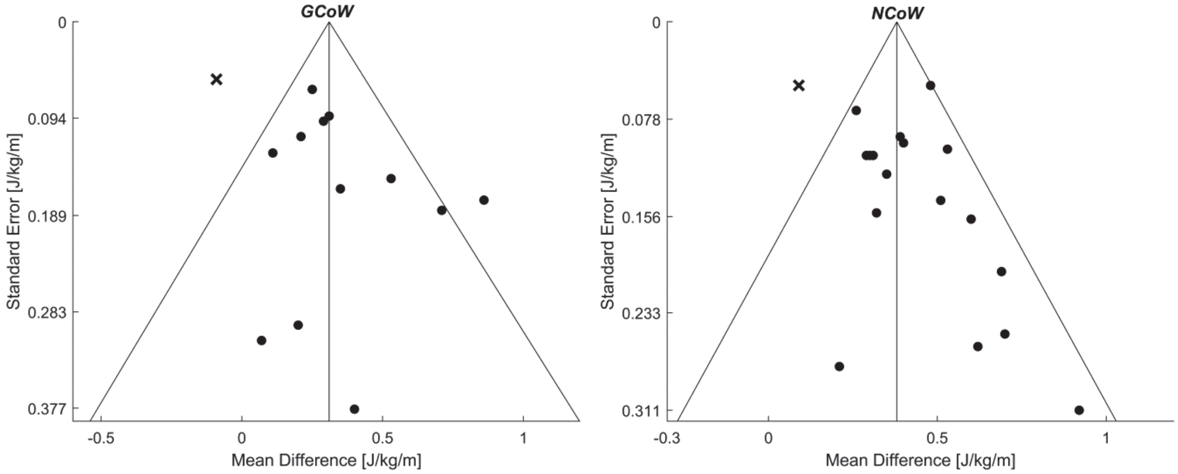

Figure C1. Funnel plot for all the studies included in the meta-analysis for GCoW (above left) and NCoW (above right). The standard errors of all the individual studies are plotted against their MD. The vertical black line in the center of the funnels represents the overall pooled MD and the two sides of the funnels represent the "pseudo" 95% CI of the overall pooled MD. The outliers are plotted automatically outside the funnels and in absence of any publication bias and/or heterogeneity, the number of studies should look symmetrical on both sides of the vertical black line. The black cross in the above figures denote the study by Gaesser et al.,[8]. That study has the lowest standard error and the highest relative weight among all the studies in our meta-analyses and is arguably the most precise on an individual level.

Appendix D – Search Strategy

The full search strategy with the query terms used for each of the four databases are detailed below.

PubMed Session Results (14 Aug 2018) 704 items

(("Energy Metabolism"[Mesh:noexp] OR "Oxidative Phosphorylation"[Mesh] OR energy metabolism[tiab] OR energy expenditure[tiab] OR metabolic cost*[tiab] OR energy cost*[tiab] OR energetic cost*[tiab] OR metabolic consum*[tiab] OR energy consum*[tiab]) AND ("Gait/physiology"[Mesh] OR "Walking/physiology"[Mesh] OR walk[tiab] OR walking[tiab] OR gait[tiab]) AND ("Aged"[Mesh] OR "Aged, 80 and over"[Mesh] OR "Geriatrics"[Mesh] OR "Geriatric Nursing"[Mesh] OR "Health Services for the Aged"[Mesh] OR elder*[tw] OR eldest[tw] OR geriatri*[tw] OR old age*[tw] OR oldest old*[tw] OR senior*[tw] OR senium[tw] OR very old*[tw] OR septuagenarian*[tw] OR octagenarian*[tw] OR octogenarian*[tw] OR nonagenarian*[tw] OR centarian*[tw] OR centenarian*[tw] OR supercentenarian*[tw] OR older people[tw] OR older subject*[tw] OR older patient*[tw] OR older age*[tw] OR older adult*[tw] OR older man[tw] OR older men[tw] OR older male[tw] OR older woman[tw] OR older women[tw] OR older female[tw] OR older population*[tw] OR older person*[tw])) NOT ("Animals"[Mesh] NOT "Humans"[Mesh])

Embase.com Session Results (14 Aug 2018) 722 items

('energy metabolism'/de OR 'oxidative phosphorylation'/exp OR 'energy metabolism':ab,ti,kw OR 'energy expenditure':ab,ti,kw OR 'metabolic cost*':ab,ti,kw OR 'energy cost*':ab,ti,kw OR 'energetic cost*':ab,ti,kw OR 'metabolic consum*':ab,ti,kw OR 'energy consum*':ab,ti,kw) AND ('walking'/exp OR walk:ab,ti,kw OR walking:ab,ti,kw OR gait:ab,ti,kw) AND ('aged'/exp OR 'geriatrics'/exp OR 'elderly care'/exp OR elder*:de,ab,ti,kw OR eldest:de,ab,ti,kw OR geriatri*:de,ab,ti,kw OR 'old age*':de,ab,ti,kw OR 'oldest old*':de,ab,ti,kw OR senior*:de,ab,ti,kw OR senium:de,ab,ti,kw OR 'very old*':de,ab,ti,kw OR septuagenarian*:de,ab,ti,kw OR octagenarian*:de,ab,ti,kw OR octogenarian*:de,ab,ti,kw OR nonagenarian*:de,ab,ti,kw OR centarian*:de,ab,ti,kw OR centenarian*:de,ab,ti,kw OR supercentenarian*:de,ab,ti,kw OR 'older people':de,ab,ti,kw OR 'older subject*':de,ab,ti,kw OR 'older patient*':de,ab,ti,kw OR 'older age*':de,ab,ti,kw OR 'older adult*':de,ab,ti,kw OR 'older man':de,ab,ti,kw OR 'older men':de,ab,ti,kw OR 'older male':de,ab,ti,kw OR 'older woman':de,ab,ti,kw OR 'older women':de,ab,ti,kw OR 'older female':de,ab,ti,kw OR 'older population*':de,ab,ti,kw OR 'older person*':de,ab,ti,kw) NOT ([animals]/lim NOT [humans]/lim)

SPORTDiscus Session Results (14 Aug 2018) 125 items

(DE "ENERGY metabolism" OR DE "AEROBIC metabolism" OR DE "ANAEROBIC metabolism" OR DE "BASAL metabolism" OR DE "CALORIC expenditure" OR TI ("energy metabolism" OR "energy expenditure" OR "metabolic cost*" OR "energy cost*" OR "energetic cost*" OR "metabolic consum*" OR "energy consum*") OR AB ("energy metabolism" OR "energy expenditure" OR "metabolic cost*" OR "energy cost*" OR "energetic cost*" OR "metabolic consum*" OR "energy consum*")) AND (DE "WALKING" OR DE "GAIT in humans" OR TI (walk OR walking OR gait) OR AB (walk OR walking OR gait)) AND (DE "OLDER people" OR DE "GERIATRICS" OR TI (elder* OR eldest OR geriatri* OR "old age*" OR "oldest old*" OR senior* OR senium OR "very old*" OR septuagenarian* OR octagenarian* OR octogenarian* OR nonagenarian* OR centarian* OR centenarian* OR supercentenarian* OR "older people" OR "older subject*" OR "older patient*" OR "older age*" OR "older adult*" OR "older man" OR "older men" OR "older male" OR "older woman" OR "older women" OR "older female" OR "older population*" OR "older person*") OR AB (elder* OR eldest OR geriatri* OR "old age*" OR "oldest old*" OR senior* OR senium OR "very old*" OR septuagenarian* OR octagenarian* OR octogenarian* OR nonagenarian* OR centarian* OR centenarian* OR supercentenarian* OR "older people" OR "older subject*" OR "older patient*" OR "older age*" OR "older adult*" OR "older man" OR "older men" OR "older male" OR "older woman" OR "older women" OR "older female" OR "older population*" OR "older person*"))

Cochrane Library Session Results (14 Aug 2018) 45 items

(("energy metabolism" or "energy expenditure" or "metabolic cost*" or "energy cost*" OR "energetic cost*" or "metabolic consum*" or "energy consum*") AND (walk or walking or gait) AND (elder* or eldest or geriatri* or "old age*" or "oldest old*" or senior* or senium or "very old*" or septuagenarian* or octagenarian* or octogenarian* or nonagenarian* or centarian* or centenarian* or supercentenarian* or "older people" or "older subject*" or "older patient*" or "older age*" or "older adult*" or "older man" or "older men" or "older male" or "older woman" or "older women" or "older female" or "older population*" or "older person*")):ti,ab,kw
